# Spatiotemporal control of genome recombination through combined FLP-Frt and GAL4-UAS technologies

**DOI:** 10.17912/micropub.biology.000089

**Published:** 2019-01-29

**Authors:** Cristina Ayuso, Peter Askjaer

**Affiliations:** 1 Andalusian Center for Developmental Biology (CABD), CSIC/JA/Universidad Pablo de Olavide, 41013 Seville, Spain

**Figure 1:**
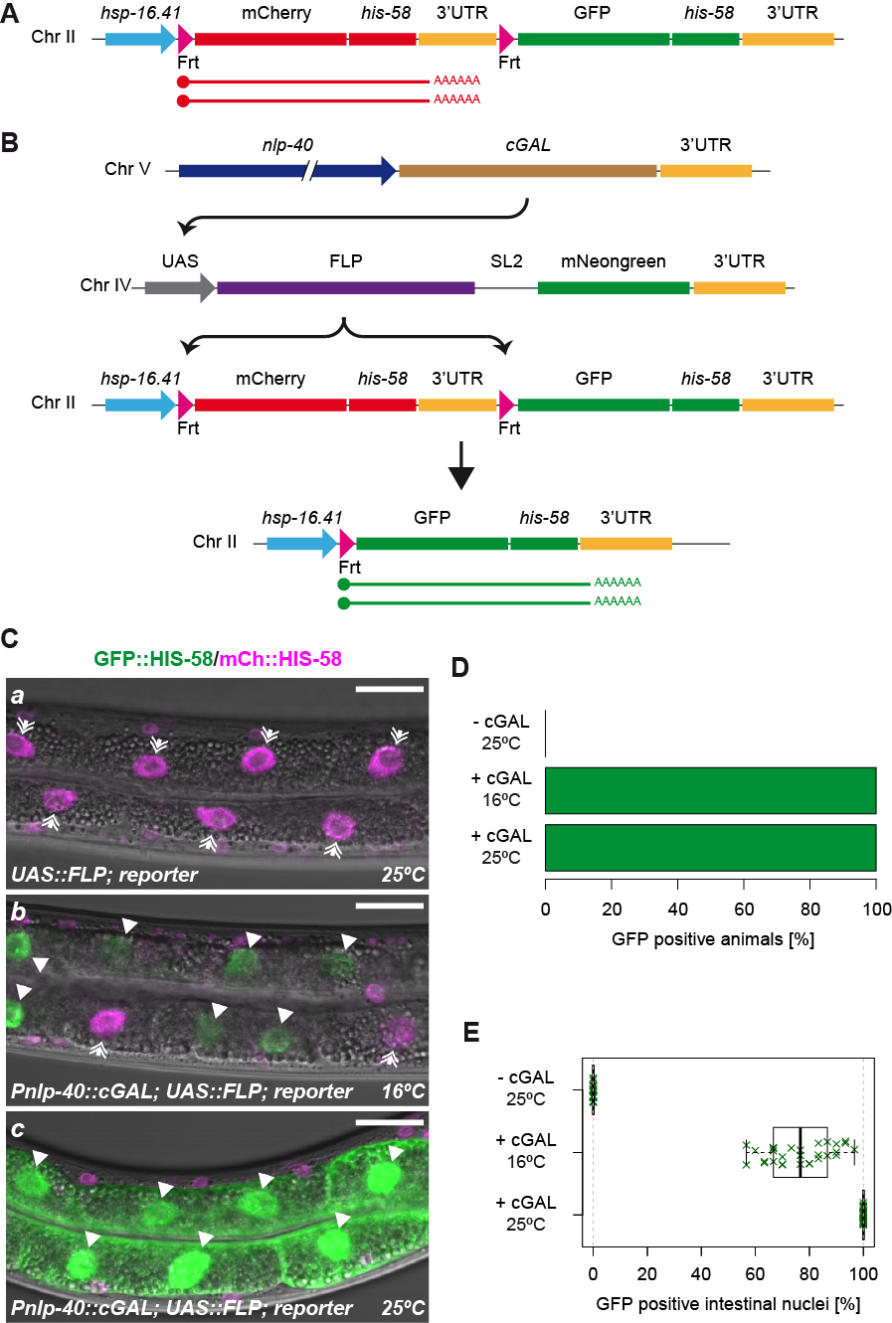
**Spatiotemporal control of genome recombination through combined FLP and cGAL activities**. (A) Reporter construct used to evaluate FLP recombinase activity. In the absence of FLP, heat induction produces red histones (mCherry::HIS-58). (B) In cells expressing cGAL from a tissue-specific promoter (e.g. *nlp-40* in the intestine), FLP is expressed and recombines the two Frt sequences. This juxtaposes the *hsp-16.41* promoter and the downstream reporter: heat induction produces green histones (GFP::HIS-58). The UAS::FLP transgene also expresses unfused mNeongreen (mNG) as marker. All transgenes are inserted into the genome: the UAS::FLP and reporter constructs are single-copy transgenes, whereas the cGAL construct is multicopy. (C) L4 larvae observed by live confocal microscopy. In the absence of cGAL (top; strain BN923), FLP is not expressed and heat induction produces mCherry::HIS-58 (double-headed arrows). In contrast, in the presence of cGAL (middle: worms grown at 16°C; bottom: worms grown at 25°C; strain BN919), most intestinal nuclei express GFP::HIS-58 (filled arrowheads). Note that cytoplasmic mNeongreen expression is clearly visible in the intestine at 25°C. Scale bar 20 µm. (D) Percentage of animals that express GFP::HIS-58 in intestinal cells after heat induction in the absence or presence of cGAL (n>100 mixed-stage animals for each condition). (E) Percentage of intestinal nuclei expressing GFP::HIS-58 after heat induction in the absence or presence of cGAL (n>20 L4s for each condition).

## Description

Several techniques are available for spatiotemporal control of genome recombination and gene expression in the nematode *Caenorhabditis elegans*. Here we report a novel tool to combine the powerful FLP-Frt and GAL4-UAS systems to increase their versality and to offer additional levels of control.

FLP is an enzyme that catalyzes recombination between two short Frt DNA sequences and is frequently used to excise genomic fragments flanked by Frt sites, thereby either activating or knocking out gene expression, depending on the experimental design (Hubbard 2014). Recently, we generated multiple strains that stably express FLP in different somatic tissues from single-copy transgenes and demonstrated that they in most cases induce recombination in ~100% of the cells of the expected tissue (Munoz-Jimenez *et al.* 2017). We subsequently constructed a strain for germline recombination to permanently knock out Frt-flanked genes or exons (Macías-León and Askjaer 2018).

The GAL4-UAS system is based on the *Saccharomyces cerevisiae* Gal4p transcription factor and its cognate DNA target called upstream activating sequence (UAS). Typically, this bipartite system includes a series of ‘driver’ strains expressing GAL4 in specific tissues and one or several strains with an ‘effector’ gene downstream of UAS repeats. Wang and colleagues from the Sternberg laboratory recently optimized the GAL4-UAS system for *C. elegans* (“cGAL”) and reported several tissue-specific cGAL drivers (Wang *et al.* 2017). Moreover, they have developed a split cGAL toolkit where the DNA binding and activation domains are expressed as individual polypeptides, thereby enabling further fine-tuning of spatiotemporal control: only when and where the two components are co-expressed they will activate the UAS::effector transgene (Wang *et al.* 2018).

We reasoned that a UAS::FLP effector strain would be a useful addition to the *C. elegans* genome engineering and gene expression toolbox. In particular, it will enable 1) recombination in tissues for which cGAL drivers have been generated, without the need to create new FLP drivers and 2) the use of intersectional promotors and split cGAL to induce recombination in cell types for which a specific promoter is not available. As proof of principle, we tested the ability of cGAL expressed in the intestine to induce UAS::FLP-mediated genome recombination.

To generate a UAS::FLP effector strain, we first excised a *15xUAS::∆pes-10* fragment from pHW394 (Wang *et al.* 2017; kind gift from Han Wang and Paul Sternberg) by digestion with *Hind*III (blunted by Klenow) and *Kpn*I and inserted it into pBN338 (Munoz-Jimenez *et al.* 2017) digested by *Not*I (blunted by Klenow) and *Kpn*I. This generated plasmid pBN449 UAS::FLP::SL2::mNG, which contains homology arms for Mos1-mediated insertion into the *cxTi10882* locus on chr IV of strain EG6700. We employed standard MosSCI procedures (Frøkjær-Jensen *et al.* 2012) to obtain the integrated strain BN908 (see strain genotypes in Reagents below). We next crossed BN908 and PS6935 (from CGC) to BN596 to obtain BN923 and BN918, respectively. Finally, BN908 was crossed to BN918 to obtain BN919.

As readout for FLP activity, we used a heat-inducible dual color reporter (Fig 1A; Munoz-Jimenez *et al.* 2017). In the absence of cGAL (strain BN923), the reporter produces only mCherry::HIS-58, demonstrating that spurious expression from the UAS::FLP is not detectable (Fig 1Ca; Fig 1D; >100 animals analyzed at 16°C and 25°C). When combined with cGAL expressed in the intestine from a *nlp-40* promoter (strain BN919), bright nuclear GFP::HIS-58 was detected specifically in this tissue, indicative of correct FLP activation and excision of the mCherry::*his-58* cassette (Fig 1B; Fig 1Cb-c). GFP::HIS-58 expression was observed in all animals both at 16°C and 25°C (Fig 1D; >100 mixed stage animals in each condition). Moreover, in animals grown at 25°C, recombination was detected in 100% of the intestinal cells (Fig 1Cc; Fig 1E; *n*=24 L2-L4 larvae), arguing that the combined activity of the UAS-GAL4 and FLP-Frt systems is very high. In animals grown at 16°C, the recombination efficiency was slightly lower with an average of 76.4% of the intestinal cells expressing GFP::HIS-58 (Fig 1Cb; Fig 1E; *n*=29 L2-L4 larvae; standard deviation = 11.7%). We propose that the temperature dependency is mainly due the characteristics of cGAL because 1) the original description of the cGAL system reported lower activity at 16°C vs 25°C (Wang *et al.* 2017), 2) we observed lower expression of cytoplasmic mNeongreen at 16°C vs 25°C (co-expressed from the FLP transgene; compare Fig 1Cb-c) and 3) direct expression of FLP from an *elt-2* promoter induces recombination in ~100% of intestinal cells at 16°C (Munoz-Jimenez *et al.* 2017). The lower activity at 16°C can potentially be overcome by producing effector strains with multiple copies of the UAS::FLP construct (Han Wang, personal communication).

In conclusion, we have developed a novel tool that enables combining the highly efficient UAS-GAL4 and FLP-Frt systems to control genome recombination and gene expression with more versatility and better precision. We provide the UAS::FLP effector strain to the community for use with existing and future cGAL drivers.

## Reagents

Plasmid pBN449 UAS::FLP::SL2::mNG.

Strain BN596 *bqSi294*[pBN154(*unc-119(+)* P*_hsp-16.41_::FRT::mCh::his-58::FRT::gfp::his-58*)] II; *bqSi577*[pBN306(*unc-119(+)* P*_myo-2_::GFP*)] IV. Available from the authors; however, if the dual color *bqSi294* effector is not needed, we recommend using BN578, which carries the same visible marker in the MosSCI locus on chr IV (*cxTi10882*) and an additional visible marker on chr II (*ttTi5605*). BN578 is available at the CGC and facilitates crosses involving MosSCI strains that carry non-visible insertions in the *ttTi5605* and *cxTi10882* sites.

Strain BN908 *unc-119(ed9)* III; *bqSi908*[pBN449(*unc-119(+) UAS::FLP::SL2::mNG*)] IV. Will be available at the CGC pending CGC approval.

Strain BN918 *bqSi294*[pBN154(*unc-119(+)* P*_hsp-16.41_::FRT::mCh::his-58::FRT::gfp::his-58*)] II; *bqSi577*[pBN306(*unc-119(+)* P*_myo-2_::GFP*)] IV; *syIs320*[P*_nlp-40_::NLS::GAL4SK::VP64* + P*_myo-2_::NLS::mCh*] V. Available from the authors.

Strain BN919 *bqSi294*[pBN154(*unc-119(+)* P*_hsp-16.41_::FRT::mCh::his-58::FRT::gfp::his-58*)] II; *bqSi908*[pBN449(*unc-119(+) UAS::FLP::SL2::mNG*)] IV; *syIs320*[P*_nlp-40_::NLS::GAL4SK::VP64* + P*_myo-2_::NLS::mCh*] V. Available from the authors.

Strain BN923 *bqSi294*[pBN154(*unc-119(+)* P*_hsp-16.41_::FRT::mCh::his-58::FRT::gfp::his-58*)] II; *bqSi908*[pBN449(*unc-119(+) UAS::FLP::SL2::mNG)*] IV. Available from the authors.
